# Multi-GPU, Multi-Node Algorithms for Acceleration of Image Reconstruction in 3D Electrical Capacitance Tomography in Heterogeneous Distributed System †

**DOI:** 10.3390/s20020391

**Published:** 2020-01-10

**Authors:** Michał Majchrowicz, Paweł Kapusta, Lidia Jackowska-Strumiłło, Robert Banasiak, Dominik Sankowski

**Affiliations:** Institute of Applied Computer Science, Lodz University of Technology, Stefanowskiego 18/22, 90-537 Lodz, Poland; pkapust@iis.p.lodz.pl (P.K.); robert.banasiak@p.lodz.pl (R.B.); dsan@iis.p.lodz.pl (D.S.)

**Keywords:** electrical capacitance tomography, heterogeneus system, distributed systems, multi-GPU computations

## Abstract

Electrical capacitance tomography (ECT) is one of non-invasive visualization techniques which can be used for industrial process monitoring. However, acquiring images trough 3D ECT often requires performing time consuming complex computations on large size matrices. Therefore, a new parallel approach for 3D ECT image reconstruction is proposed, which is based on application of multi-GPU, multi-node algorithms in heterogeneous distributed system. This solution allows to speed up the required data processing. Distributed measurement system with a new framework for parallel computing and a special plugin dedicated to ECT are presented in the paper. Computing system architecture and its main features are described. Both data distribution as well as transmission between the computing nodes are discussed. System performance was measured using LBP and the Landweber’s reconstruction algorithms which were implemented as a part of the ECT plugin. Application of the framework with a new network communication layer reduced data transfer times significantly and improved the overall system efficiency.

## 1. Introduction

**Figure 1:**
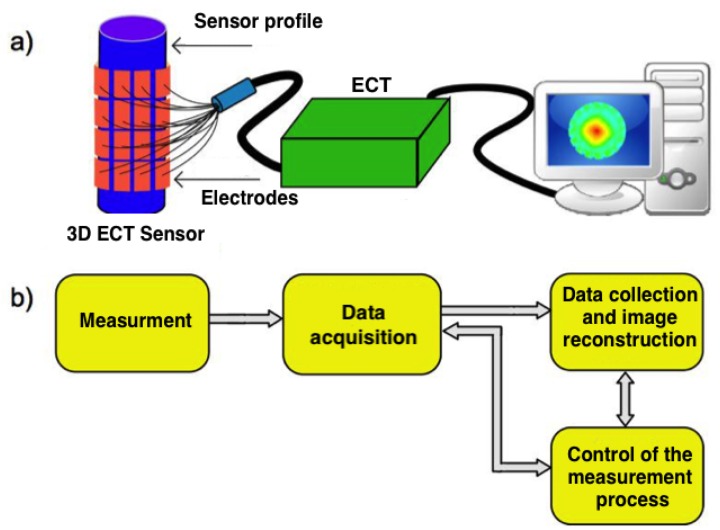
Diagram of the measuring system in the electrical capacitance tomography: (**a**) ideological— constructional, (**b**) block—functional.

Electrical capacitance tomography (ECT) is a measurement technique that can be used for non-invasive monitoring of diverse industrial processes in 2D [1,2], 3D [3] and even 4D dynamic mode [4]. ECT is performing the task of imaging of materials with a contrast in dielectric permittivity by measuring capacitance from a set of electrodes placed around the investigated object. A fundamental ECT measurement setup consisting of the ECT sensor, ECT tomograph and a computer is shown in Figure 1a. In ECT measurement cycle the following phases can be distinguished: capacitance measurement, data acquisition (signal conditioning and analog to digital conversion), data collection, and finally calculating of electrical permittivity distribution, which is called image reconstruction. The entire measurement process is controlled automatically by a computer system (Figure 1b). Accuracy of ECT imaging is determined by hardware as well as by software and both of these factors are taken into consideration while new measurement setup is designed. Sensor resolution is limited by the number of electrodes and their size. In 3D ECT usually three or four rings with 8, 12 or 16 electrodes are used. Reducing the measurement time is limited by time of capacitance measurement. Also, data acquisition and processing have a significant impact on accuracy and time limits.

In order to achieve a high quality tomographic image [5], complex reconstruction algorithms performing many matrix calculations have to be applied [6,7,8,9]. However, choosing an optimal reconstruction algorithm and its implementation depends on an application. In medicine or material science, where an internal object or dangerous material crack [10] needs to be detected, the most important feature of the algorithm is accuracy and precision of image reconstruction, while time of data processing is not so crucial. In dynamic measurements of fast changing media, time of measurement and data processing is equally or even more important parameter than accuracy. Therefore different solutions accelerating these calculation have been reported in the past, especially dealing with parallel computing [11,12,13,14,15], sparse matrices and Finite Elements Method [16], Fourier-based sparse representations [17], neural networks approach [18,19], fuzzy logic [20] or field-programmable gate array (FPGA) implementation [21]. Usually a compromise between accuracy and rapidity of ECT measurement must be taken. While for control purposes determination of some important process parameters can be sufficient, fast and accurate 3D process visualisation is a challenging task.

In this work the authors concentrate on parallel computing in distributed system as computational capabilities of single PC proved to be one of the main limiting factors for applying 3D ECT for on-line industrial process monitoring, such as for example oil-gas flow [3] or silo flow [22] monitoring.

Distributed systems are one of the most important technological achievements in recent years [23,24], which have had a significant impact on the development of modern computing. The scope of distributed systems applications in everyday life is very wide [25,26], from local systems such as cars, ships, aircraft to global systems of millions of nodes used for data processing services; from simple built-in systems consisting of very small and simple sensors to those containing powerful computational components [27]; from built-in systems to those that support advanced interactive user interfaces.

A distributed system can be defined as one in which the hardware components or software on the computers in the network communicate and coordinate their actions only through the transmission of messages [28,29]. This simple definition covers a whole range of systems in which networked computers can be successfully used to perform tasks faster or more accurately than a single unit. The main motivation for the construction and use of distributed systems is the desire to separate resources [30,31,32]. The concept of “resources” that can be effectively shared on a computer network includes both hardware components such as disks and printers, and software such as files, databases, and other objects such as video streams that are transmitted from a digital video camera or a voice signal transmitted between mobile phones and stations.

In this paper, a novel heterogeneous, multi-graphics processing unit (GPU), multi-node distributed system is proposed, with a framework for parallel computing and a special plugin dedicated to ECT. The system’s architecture and features are presented, and also parallel algorithms of ECT data processing and image reconstruction. Test results comparing the developed system with new network communication layer are compared to the authors’ earlier solutions based on the Xgrid platform [13,33]. Finally, the results of the latest system modification and optimization are presented and compared to the previously developed solutions. [34,35].

## 2. Image Reconstruction in ECT

The scheme of image synthesis in electrical capacitance tomography is called image reconstruction. It is based on solving the so called inverse problem [36], in which the spatial distribution of electric permittivity is approximated from the measured values of capacitances between the sensor electrodes. Inverse problem in ECT is ill posed due to the small number of capacitance C measurements in relation to number of pixels in reconstructing image. This results a poor spatial resolution of ECT technique.

Inverse problem is nonlinear and therefore two main groups of image reconstruction algorithms can be distinguished: nonlinear and linear. Non-linear algorithms are more accurate but slower [16]. Linear algorithms use approximate linear model, which is less accurate but simple and useful in engineering praxis. These algorithms are used predominantly for monitoring fast-varying industrial processes, like oil-gas flows in pipelines [3] or gravitational flows and discharging of silo [22].

One of the most used reconstruction algorithms in ECT is linear back-projection (LBP). Even though it is characterized by low spatial resolution, it is not as computationally complex as other solutions. Moreover, there is still active research on improving its characteristics [37]). LBP is based on the following Equation [8,38]:

(1) $$\begin{array}{c} \varepsilon =S^{T}C_{m} \end{array}$$

where:

ε—electric permittivity vector (output image),
$S^{T}$—sensitivity matrix, transposed
$C_{m}$—capacitance measurements vector.

The Landweber’s algorithm is a more accurate, iterative algorithm, which is based on the following Equation [8,38]:

(2) $$\begin{array}{c} \varepsilon_{k+1}=\varepsilon_{k}-\alpha S^{T}\left(S\varepsilon_{k}-C_{m}\right) \end{array}$$

where:

$\varepsilon_{k+1}$—image obtained in current iteration,
$\varepsilon_{k}$—image from the previous iteration,
α—convergence factor (scalar),
$S^{T}$—sensitivity matrix, transposed,
S—sensitivity matrix,
$C_{m}$—capacitance measurements vector.

Computation of sensitivity matrix S for ECT tomography incorporates a sensitivity model based on an energy of electric field accumulated in a 3D ECT sensor space. This energy can be calculated as the energy of capacitor or as the energy of electric field:

(3) $$\begin{array}{c} W_{C}=\frac{1}{2}U^{2}C \end{array}$$

(4) $$\begin{array}{c} W_{E}=\frac{1}{2}\int_{\Omega }\varepsilon (x,y,z)\vec{E}\left(x,y,z\right)\cdot \vec{E}\left(x,y,z\right)d\Omega \end{array}$$

where: $W_{C}$ is an electric field energy accumulated in capacitor with capacity C and with applied voltage U, $W_{E}$ is an energy of electric field E with spatial electric permittivity distribution ε(x,y,z). Of course both equations describe the same energy therefore they can be compared together:

(5) $$\begin{array}{c} \frac{1}{2}U^{2}C=\frac{1}{2}\int_{\Omega }\varepsilon (x,y,z)\vec{E}\left(x,y,z\right)\cdot \vec{E}\left(x,y,z\right)d\Omega \end{array}$$

and consequently:

(6) $$C=\frac{1}{U^{2}}\int_{\Omega }\varepsilon (x,y,z){\vec{E}}^{2}\left(x,y,z\right)d\Omega$$

The sensitivity value of any point j∈Ω describes the connections between changes of spatial permittivity distribution in that point and resulting changes of the capacity i, as it can be expressed by followed equation:

(7) $$S_{i,j}=\frac{\delta C_{i}}{\delta \varepsilon_{j}}$$

Combining both Equations (6) and (7) and omitting i index which is connected with measurement sequence we can get:

(8) $$\begin{array}{c} S_{j}=\frac{1}{U^{2}}\frac{\delta (\int_{\Omega }\varepsilon (x,y,z){\vec{E}}^{2}\left(x,y,z\right)d\Omega )}{\delta \varepsilon_{j}} \end{array}$$

However it is still not defined what $\varepsilon_{i}$ means and how it is related to the spatial electric permittivity distribution function ε(x,y,z). Typically when finite elements modeling for ECT is used the function ε(x,y,z) can be defined as constant value inside each point j∈Ω. Therefore Equation (8) can be rewritten as:

(9) $$\begin{array}{c} S_{j}=\frac{1}{U^{2}}\frac{\delta (\int_{\Omega }\varepsilon_{j}{\vec{E}}^{2}\left(x,y,z\right)d\Omega )}{\delta \varepsilon_{i}} \end{array}$$

Assuming that voltage U equals to a difference between potentials $\varphi_{el1}$ and $\varphi_{el2}$ applied to electrodes el1 and el1 and expanding Equation (8) we can compute the sensitivity matrix S using formula:

(10) $$\begin{array}{c} S_{i,j}\left(x,y,z\right)=\frac{1}{\varphi_{el1}\cdot \varphi_{el2}}\frac{\delta (\int_{\Omega }\varepsilon_{j}\left(x,y,z\right)\vec{E_{el1,i}}\left(x,y,z\right)\cdot \vec{E_{el2,i}}\left(x,y,z\right)d\Omega )}{\delta \varepsilon_{j}} \end{array}$$

where $S_{i,j}\left(x,y,z\right)$ is a sensitivity value for point j∈(x,y,z) while i-th capacitance measurement between electrodes ${el}_{1}$ and ${el}_{2}$ formed, $\varphi_{el1}$ and $\varphi_{el2}$ are potentials applied to electrodes ${el}_{1}$ and ${el}_{2}$ respectively. $\vec{E_{el1,i}}\left(x,y,z\right)$ and $\vec{E_{el2,i}}\left(x,y,z\right)$ are electric field vectors, $\varepsilon_{j}$ is a permittivity value for the point j∈(x,y,z).

Potentials $\varphi_{el1}$ and $\varphi_{el2}$ can be determined numerically using FEM modelling according to general formula:

(11) $$\begin{array}{c} \varphi =Y^{-1}F \end{array}$$

where φ is a sought distribution of the electric field—represented by the spatial distribution of nodal potential—partial solution of the forward problem in capacitance tomography;

Y is a transformation matrix, built according to the geometric dependencies of sensor model mesh and Neumann boundary conditions;

F is the right-hand side forces vector, defining the given Dirichlet boundary conditions

Electric field distribution φ can be further used for Gauss Law based inter-electrode capacitance computations:

(12) $$\begin{array}{c} C_{e1e2}=\frac{{\;\int \int \int \;}_{\Omega }\varepsilon \left(x,y,z\right)\mathrm{grad}\left[\varphi \left(x,y,z\right)\right]d\Omega }{\varphi_{e1}-\varphi_{e2}} \end{array}$$

where:

$C_{e1e2}$—Capacitance between electrodes *e*1 and *e*2
ε(x,y,z)—distribution of electric permittivity in domain Ω
φ(x,y,z)—distribution of electric potential in domain Ω
$\varphi_{e1}$—electric potential applied to electrode *e*1
$\varphi_{e2}$—electric potential applied to electrode *e*2
x,y,z—Cartesian coordinates in domain Ω

The sensitivity matrix is an important part of the image reconstruction algorithms and has to be recalculated and tweaked for every sensor and specific visualization task. In some applications it is necessary to update it regularly and use non-linear reconstruction algorithm [16]. However, in this work only the linear Landweber’s algorithm is considered, so the sensitivity matrix calculation is a one-time process, after which the matrix is then distributed to each computing node. It is then treated as a constant in computations and does not contribute to the data transfer time during the reconstruction.

In the case of the Landweber’s algorithm each iteration improves the overall quality of the output image. The more iterations are performed the better the image quality is obtained, however the overall time of computations increases. As a result acceleration of image reconstruction process is a very important issue. Nowadays, industrial applications of 3D electrical capacitance tomography are mainly limited by the two combined factors: image quality and computational complexity of image reconstruction process [39].

**Figure 2:**
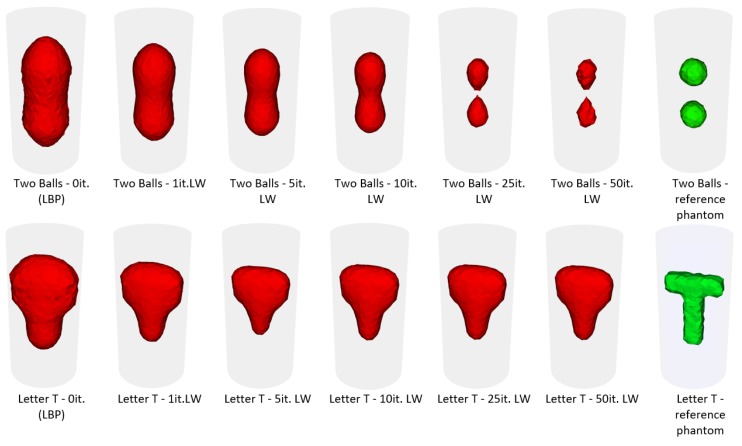
Dependency between 3D image reconstruction quality and number of Landweber’s algorithm iterations (it. LW) for two example objects (simple two balls and challenging T letter shape). From the left to the right : (in red) LBP (0it. LW), 1it LW, 5it LW, 10it LW, 25it LW, 50it LW, reference phantom object (in green).

The difference in quality of the reconstructed image, caused by the difference in number of Landweber’s algorithm iterations that were possible, can be seen in Figure (Figure 2), which shows reconstruction quality improvement rate for given iterations number, using two examples (two simple balls and challenging T letter shape).

Image reconstruction using deterministic methods requires execution of a large number of basic operations of linear algebra, such as transposition, multiplication, addition and subtraction [34,40]. Matrix calculations for a large number of elements is characterized by a high computational load [41]. Moreover, their computational complexity class is polynomial. For example, matrix multiplication is an algorithm of O($n^{2+\varepsilon }$) class [42], for any constant ε > 0, which means that n-fold increase in the multiplied matrix dimensions will $n^{2+\varepsilon }$ fold increase the execution time. Matrix multiplication is a key operation in ECT imaging and therefore some researchers decided even to build a custom hardware for this purpose to speed up computations [43].

In this work, an approach based on computations on GPUs is proposed, as it allows for high flexibility and relatively low application costs. Algorithms of ECT image reconstruction in a distributed multi-node, multi-GPU environment were developed and tested. Two image reconstruction algorithms were implemented: the LBP and the Landweber’s algorithm.

Due to the nature of the Landweber’s algorithm it is necessary to exchange the data ($\varepsilon_{k+1}$) in every iteration, so its parallelization is a challenging task. The main concept of our implementation of the Landweber’s reconstruction in distributed system will be discussed in details in Section 4.

## 3. Developed Distributed System

As a result of the earlier studies [13,41] a new distributed system dedicated to ECT computations has been developed. The system is specifically designed to accelerate matrix computations that are a crucial part of reconstruction algorithms used in ECT [16]. The previously developed solution was based on the Xgrid platform, used as a network layer. However, the analysis of this system showed the limitations of this solution, and the main conclusion from the previous research [33] was that the new software for the system should be developed and new network communication should be implemented.

### 3.1. Design Assumptions

The use of a heterogeneous system for distributed computing in ECT required the solution of series of tasks without which the proposed system would not work properly. The most important of these are as follows:

- Division of matrices between nodes
- Basic operations of linear algebra (transposition, addition, subtraction, multiplication)
- Data transfer between nodes
- Planning and division of tasks
- Support for heterogeneous devices
- Support for calculations using graphics cards
- Support for modern multi-core processors as a set of devices
- Possibility to extend existing solutions with pseudo inheritance from implemented layouts

### 3.2. Implementation

**Figure 3:**
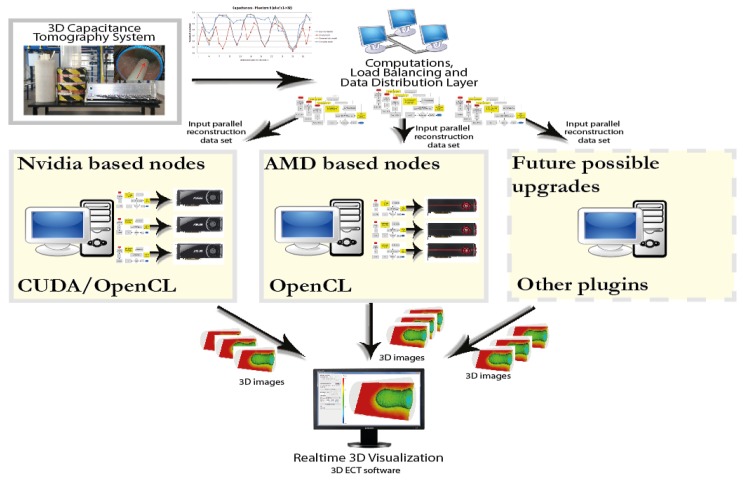
Schematic of a designed heterogeneous distributed computing system comprising graphics cards.

The architecture of the designed system is shown in Figure 3.

The system contains both GPU and CPU based computing nodes for performing general purpose computation. It has an open structure and is designed to utilize currently sold hardware, but also for easy extension in the future with plugins and other upgrades.

In our new solution a special framework called KIS digital computing (KISDC), where KIS is an abbreviation of the Polish name of the Computer Engineering Department, was designed and built that provides software tools needed both for the system architecture expansion and new algorithms development and implementation in a distributed heterogeneous environment [35].

**Figure 4:**
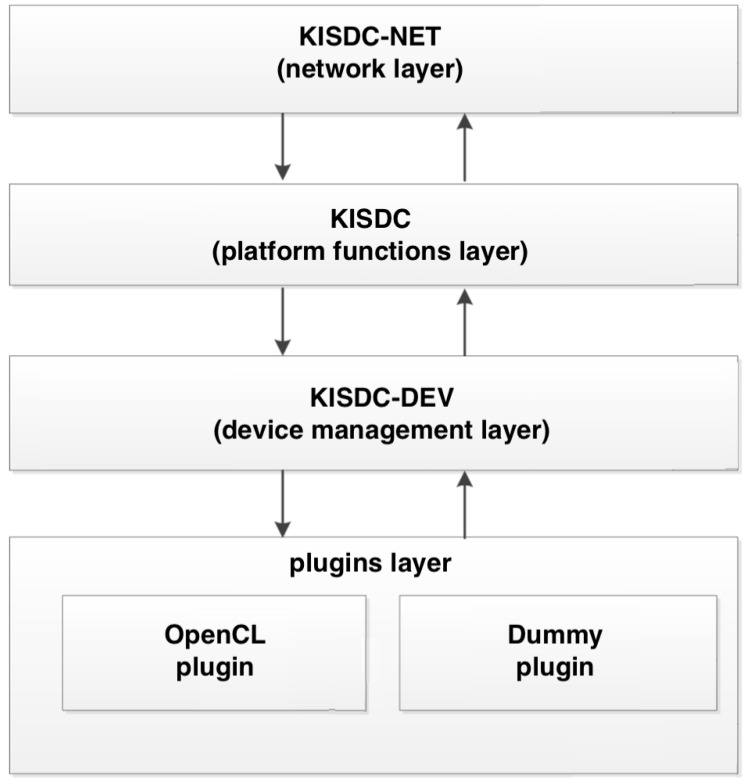
Block diagram of different layers in KIS digital computing (KISDC) platform.

The proposed approach allows for a greater flexibility of the developed solutions, provides tools for their easy testing and enables further acceleration of ECT image reconstruction. The system was designed as a modular, layered architecture (Figure 4). This approach allows limiting the dependencies between the individual modules. Moreover, thanks to this architecture, it is possible to abstract the compute devices using KISDC-DEV module. It is an abstraction and management layer that hides the type of the underlying hardware from the user and makes all the algorithms written using the provided application programming interface (APIs) hardware-agnostic.

Expansion of the computing power of the system is possible through the use of “plug-in” architecture (by adding support for new devices, such as FPGAs). The basic operations of linear algebra were implemented in the system as a set of functions in the form of an API.

**Figure 5:**
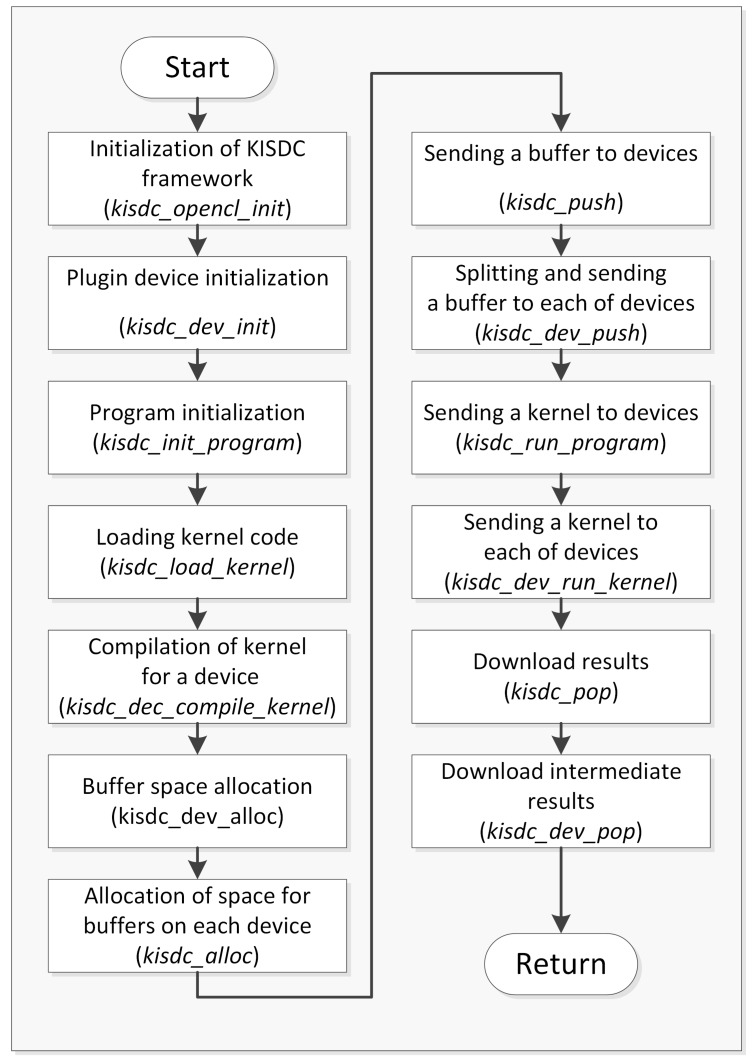
Activity block diagram for performing calculations using the KISDC platform—each block contains a name of activity and a name of corresponding function in KISDC framework realizing this activity (in parenthesis).

The framework was designed to ensure an efficient use of the computing power of all the devices present in the nodes. This architecture is scalable and allows users to expand the capabilities of the system by adding more nodes. The above assumptions pose many challenges in the architecture of the system itself, but their application makes it straightforward to use the environment to speed up computations in existing projects, thus testing and developing new distributed algorithms is much faster. As shown in the activity diagram (Figure 5), the process of performing calculations using the KISDC system allows for much more flexibility in the number and type of devices used.

**Figure 6:**
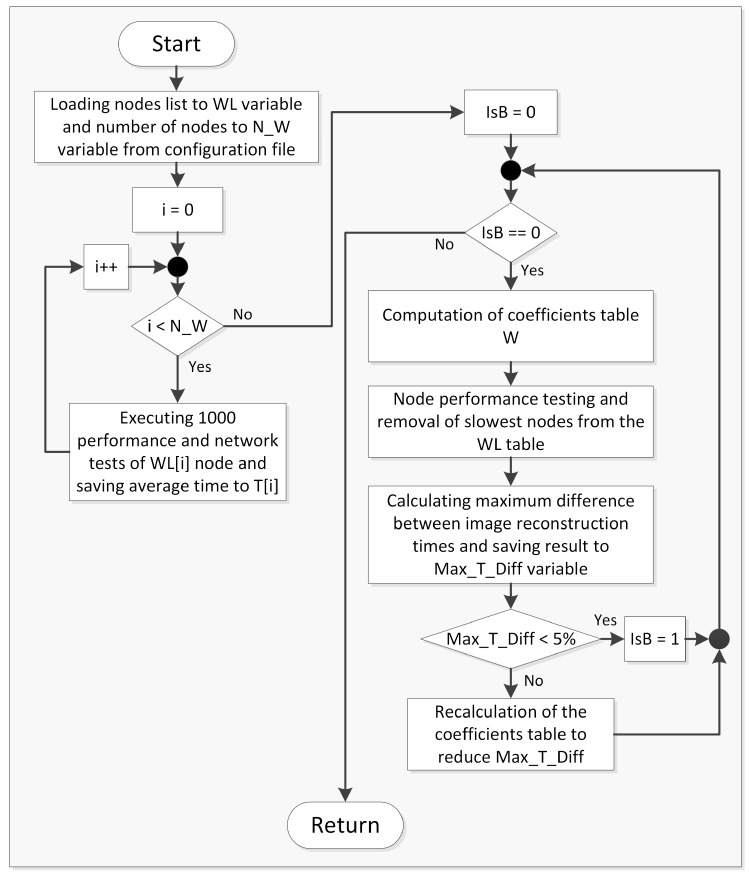
Flow diagram for coefficients computation algorithm for KISDC platform, where WL is array of nodes, N_W is number of elements in WL array, W is an array of computed coefficients, T is an array of average time for each node, Max_T_Diff is maximal time difference between nodes, IsB is bolean flag indicating if recalculation (false value) is necessary.

The system is under active development and is modified according to the new demands. As part of the latest update a special algorithm was designed that computes an array of performance coefficients (Figure 6) which is then used to optimize the utilization of the system. In the proposed solution coefficients of data distribution are evaluated in experimental way. As part of this algorithm (which is used in setup phase of KISDC platform) special benchmark procedure has been developed (Figure 6), which allows to choose the coefficient values according to the current hardware configuration and to the system working conditions.

**Figure 7:**
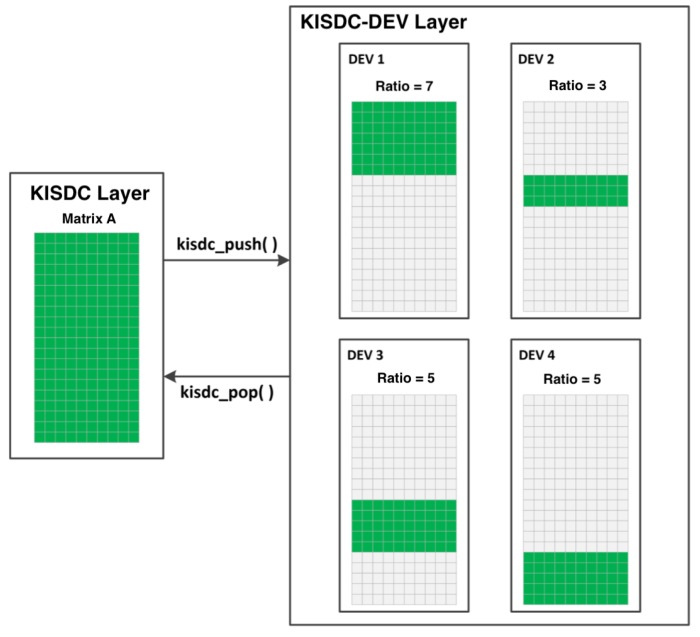
Block diagram of data division between devices on each node, kisdc_push function is responsible for transferring data to devices whereas kisdc_pop transfer data from devices.

Computed coefficients are used in the device management layer to distribute the data between devices on each node (Figure 7). Experimental method of these coefficients evaluation is applied as well for data distribution between devices on each node as well between computers in distributed system. An analogous algorithm to this presented in Figure 6 and benchmark are used for coefficients computation for data distribution between computers.

The KISDC architecture aids in the development of image reconstruction algorithms in electrical capacitance tomography, as it simplifies their implementation for various hardware configurations in a distributed system. As shown in the previous section, the process of performing calculations application of the KISDC system allows for much more flexibility in the number and type of devices used than the OpenCL library.

In order to take full advantage of the distributed nature and the heterogeneity of the system, a new solution has been developed for performing computations on big sets of data. This problem is especially prominent in case of 3D ECT, where the size of used matrices is significant. When using GPUs as computational devices the memory available is usually much less than system memory (RAM). When the computational load is proportionally divided between the devices it is possible to only send necessary data to each device. Moreover, for some algorithms and compute kernel implementations, the data split can be done on matrix basis, i.e., the data will be divided between the devices in a way that will be hidden from the algorithm, based only on the available memory and computing capabilities of the nodes.

Furthermore, other minor optimisations of the algorithm have been implemented recently, what will be more accurately described in Section 5. The whole system software has been developed in C programming language with the use of object oriented programming paradigm, dynamic data structures and OpenCL libraries. Moreover, in order to guarantee correctness of the obtained computation results, we make sure that all devices present in the system are fully compliant with IEEE754-2008 standard, describing floating-point arithmetic. We also use techniques of increasing the precision and decreasing rounding errors, like utilizing hardware accelerated fused multiply add (FMA) operations, wherever possible.

## 4. Parallel Algorithm of ECT Image Reconstruction in Distributed System

**Figure 8:**
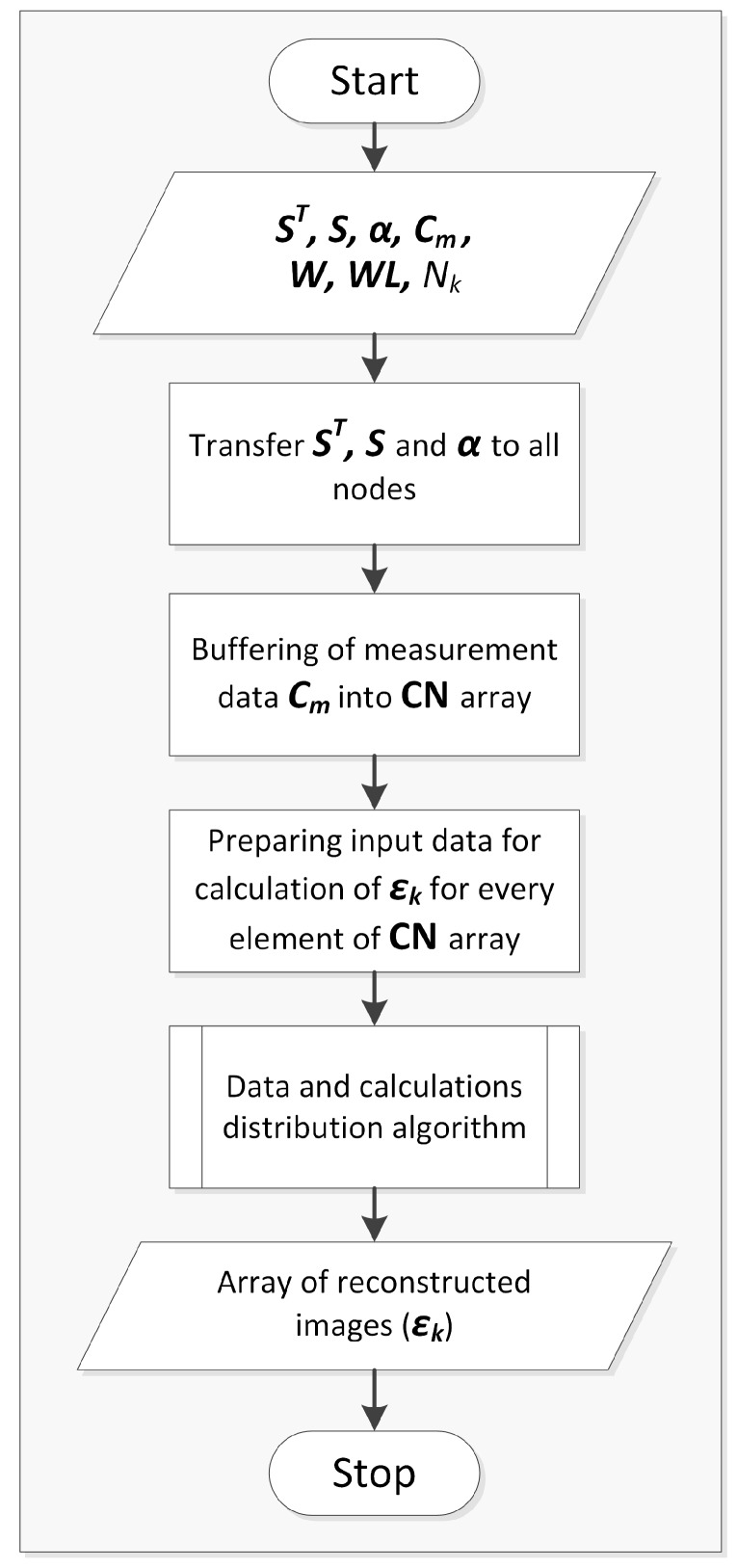
General schema of distributed Landweber’s algorithm with data buffering, where $\varepsilon_{k+1}$ is image obtained in current iteration, $\varepsilon_{k}$ is image from the previous iteration, α is convergence factor (scalar), $S^{T}$ is sensitivity matrix transposed, S is sensitivity matrix, $C_{m}$ is capacitance measurements vector, **CN** is an array of capacitance measurements vectors, ***WL*** is an array of nodes, W is an array of computed coefficients, and Nk is a number of elements in ***W*** array.

The main concept of the Landweber’s algorithm (Equation (2)) implementation in a distributed system is shown in Figure 8. Matrix S is calculated from Equation (10) as it was explained in Section 2. Due to the high density meshes needed in this case to obtain sufficient computing accuracy for 3D ECT large number of numerical operations of high computational complexity has to be performed to solve this task. Parallel distributed algorithms developed for this purpose using the Finite Element Method, sparse matrices and multiple GPUs are described in the Authors’ earlier work [16]. Matrices S and $S^{T}$ are calculated once at the beginning and remain constant, so they are sent only once to compute nodes before the other calculations. This is very important for the whole distributed system performance, because sensitivity matrices are big matrices, a few orders of magnitude bigger than measurements or output image vectors. Therefore they transferring over a LAN would have a huge impact on the system performance and would slow down the calculation process.

The next key problem is data distribution between the nodes in heterogeneous distributed system (Figure 3). In most 3D ECT systems measurement data are collected with higher frequency than they can be reconstructed. Moreover, because of the asynchronous nature of the developed solution, based on the commissioning of tasks to local GPUs using OpenCL technology, as well as remote computing nodes, delays can accumulate, therefore there is a need for their elimination by buffering systems. Measurement data C are buffered in a memory in $C_{m}$ array and they need to be prepared for $\varepsilon_{k}$ calculation before sending to the compute nodes. In the case of data distribution between computers larger amount of data containing full data frames are grouped and buffered in CN array for sending through the computer network. Within the computers smaller amounts of data are distributed between the compute nodes. Algorithms of data distribution between computers or compute nodes are based on experimentally determined coefficients corresponding to the available memory and computing capabilities of the nodes, as it was described in Section 3 and presented in Figure 6. These coefficients are also computed once for a specific hardware configuration.

In the first iteration, approximated electrical permittivity distribution $\varepsilon_{0}$ is calculated from LBP algorithm (Equation (1)), then next $\varepsilon_{k+1}$ vectors are calculated from Equation (2) in distributed system.

**Figure 9:**
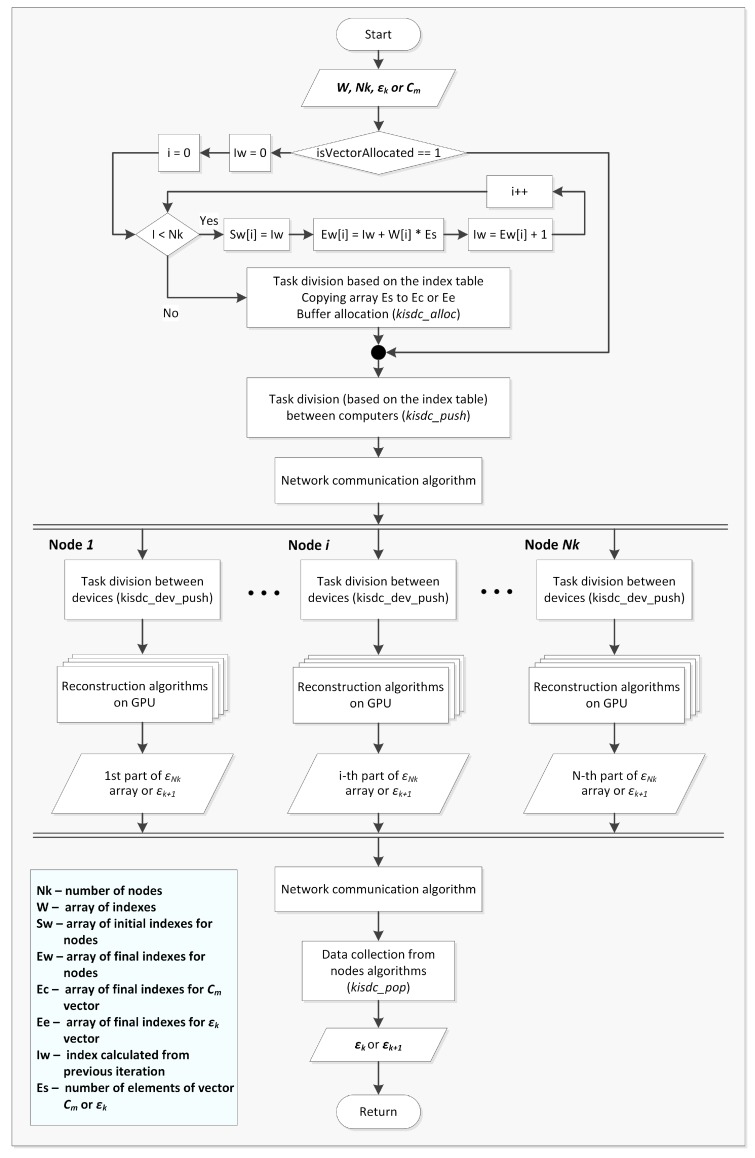
Flow diagram for data and calculations distribution algorithm.

Flow diagram of the algorithm for data and calculations distribution is shown in (Figure 9). The algorithm has been designed, implemented and optimized from the start as a solution suited to heterogeneous multi-thread CPUs and multi-GPUs distributed systems. CPUs are used for memory allocation, task division and then management of data collection from compute nodes, while GPUs are used for linear algebra operations on matrices. In order to split data according to devices, as it was shown in Figure 7, computational capabilities array of coefficents is used to calculated start (Sw) and end indeces (Ew) for data division. Due to the specific nature of the computations on GPU the most optimal solution is to start a separate thread for each GPU in the system, that are synchronised when reading the results.

The main idea of the developed algorithm is, that each GPU inside the compute node calculates the solution to the Landweber’s algorithm for a few picture elements, by assigning to each GPU calculations specific to the selected part of image, where the whole multi-GPU system computes the result of a single image reconstruction. In other words all computations of Landweber’s algorithm iterations are performed by GPUs whereas rest of the system is responsible for data transfer and division. This approach allows for more precise control of tasks allocation. This in turn enables its use in distributed systems with a high degree of heterogeneity.

Main problem of real-time image reconstruction are delays caused by computation, nevertheless data sending is also a very important issue since it introduces additional delays and hinders the whole process. Therefore, both these aspects have been tested while the system was developed. Problems of optimal hardware configuration in heterogeneous multi-CPU multi-GPU system were considered more detaily in our earlier publications [13,15]. In this work we focused more on communication issue and testing new developed network layer.

## 5. Test Results

All tests were conducted for the same hardware configuration consisted of two nodes of high computing power, using eight thread Intel i7 930 CPUs and Nvidia GPUs (Tesla S1070 + Tesla C2070 compute devices in the first and dual GeForce GTX 570 graphic cards in the second node), connected via 1 Gigabit LAN. The first part of this section is related to network layer testing. The next part deals with a performance of the whole new developed distributed system and its comparison to the previous system version. Finally, possibility of industrial process monitoring is discussed.

### 5.1. Network Layer Performance

By creating a network layer the existing solutions and network protocols can be used or the own ones can be developed. In the previously built distributed system a ready-made Xgrid platform was applied as a network layer [33].

In this work the author’s KISDC system with KISDC-NET network layer was designed and implemented. While designing the KISDC-NET layer, the existing network protocols were applied and tested in advance in order to choose the best solution.

The network characteristics of the previously developed solution based on the Xgrid system was compared with the new system using other data distribution protocols: hypertext transfer protocol (HTTP) [44], file transfer protocol (FTP) and server message block (SMB).

**Figure 10:**
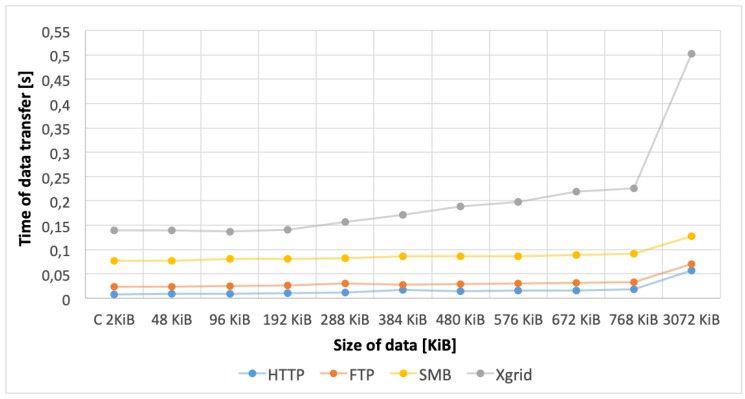
Comparison of average data transmission times for different network protocols.

**Figure 11:**
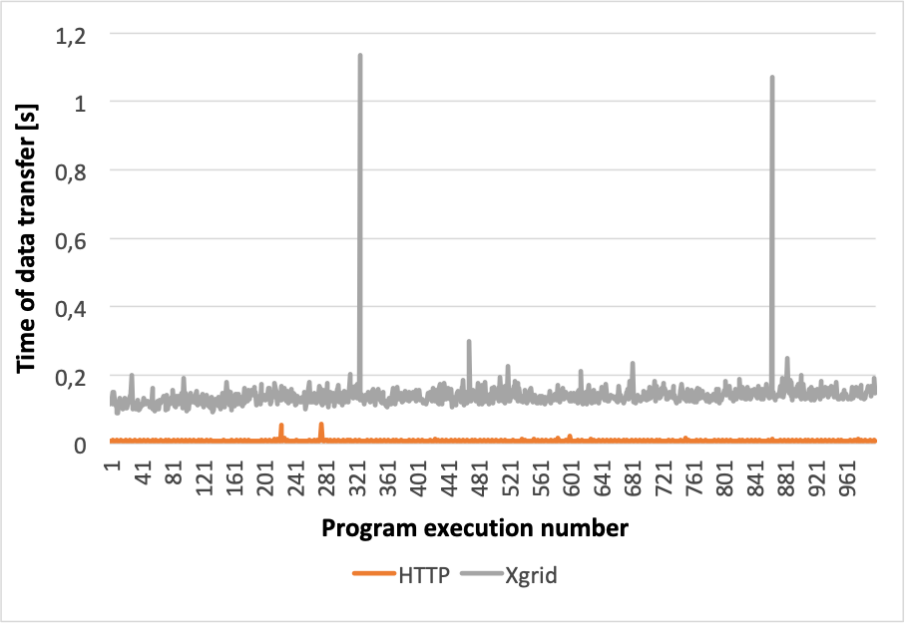
Graph showing comparison of results for hypertext transfer protocol (HTTP) and Xgrid platform for configuration with two computers and measurement data vector (C 2 KiB).

**Figure 12:**
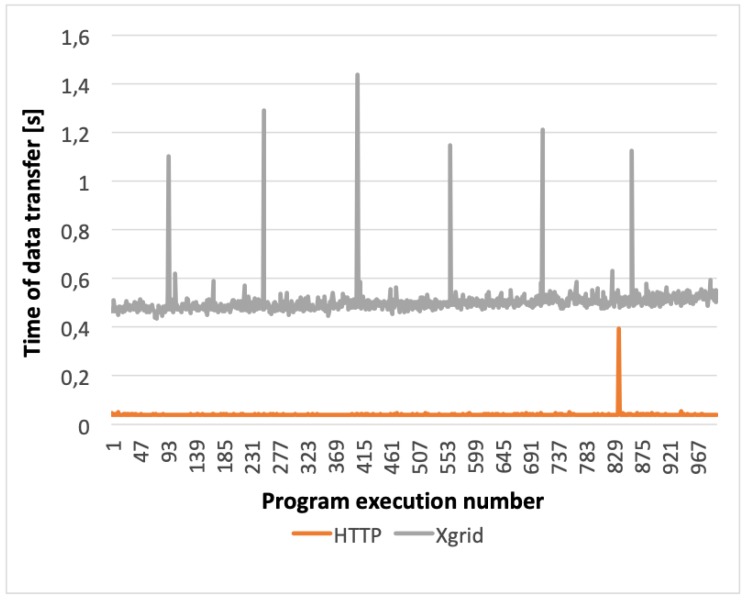
Graph showing performance comparison for HTTP protocol and Xgrid platform for two-computer configuration and 3072 KiB image data vector.

Based on the obtained results (see Figure 10) it can be stated that the use of each of the tested protocols ensured a shorter data transfer time than the Xgrid-based solution, although the best results were achieved for the HTTP protocol. Therefore, HTTP protocol was selected as the best one for the KISDC-NET. More accurate comparisons of HTTP and the Xgrid-based solutions are shown in Figure 11 and Figure 12. Average data transfer time for Xgrid was about 25 times longer than for HTTP (0.189 s and 0.008 s for 2 KiB and 0.481 s and 0.019 s for 3072 KiB respectively). It can also be noted that for Xgrid-based solution random disturbances in data transfer occurred frequently. They are visible in Figure 11 and Figure 12 as spikes with much longer than average data transfer time. For the HTTP protocol, these spikes occurred significantly less frequently and have many times smaller magnitude both for C and image vectors.

The comparison of the data transfer and communication times between the nodes shown in Figure 10 indicates very clearly that the use of HTTP protocol ensured much better results than the Xgrid platform. Already for the measurement data vector (2 KiB), it is evident that using the HTTP protocol allowed for significant reduction of the total image reconstruction time. Moreover, for the highest-resolution (3072 KiB) image vector, the use of HTTP allowed to improve significantly data transmission in a distributed environment compared to the results obtained with the Xgrid platform in the same configuration. In all cases data transmission was performed much faster in KISDC than on Xgrid platform.

### 5.2. System Performance

Both distributed systems, one based on the Xgrid platform and the KISDC, have been extensively tested and compared. In each test case the hardware configuration was the same as it was specified at the beginning of this section.

**Figure 13:**
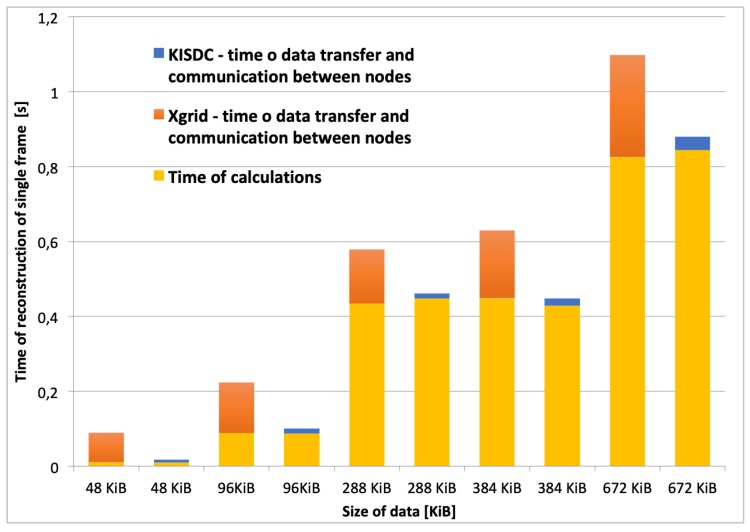
Comparison of image reconstruction time in Xgrid and KISDC systems for dual computer configuration using 400 iterations of Landweber’s algorithm.

The comparison of times of a single frame reconstruction in the two node system are shown in Figure 13. Yellow color represents calculation time (the same for the both systems), blue color is related to data transfer time for the KISDC system, and orange color denotes data transfer time for the Xgrid system. For each of the analyzed data sizes, the speed up of image reconstruction expressed in the number of reconstructed frames per second was noted. The most significant relative acceleration was achieved for 48 KiB and 96 KiB image vectors.

**Figure 14:**
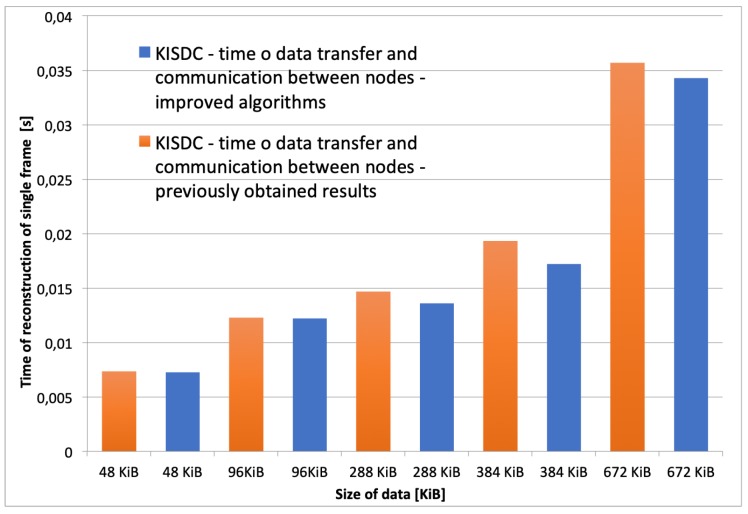
Graph showing a difference in time of image reconstruction between the improved version of the algorithm over previously obtained results.

In the recent system version a few optimizations of the algorithm have been implemented. The size field in the 4 byte header has been removed and replaced with single byte *Enum*, with defined values for all data sizes that the system supports. Moreover, calculation of $\alpha S^{T}$ is performed only once on the system start instead of in every iteration of the algorithm. Both versions of the distributed Landweber’s algorithm supporting frame buffering (Figure 8) were tested. The results are presented in Figure 14). It should be noted that implementation of the optimizations in calculations and data transfer layers allowed for significant reduction of the total image reconstruction time in comparison to previously reported results [35].

### 5.3. Monitoring of Industrial Processes

In order to monitor fast changing industrial processes in a satisfactory manner it is necessary to achieve high image quality at high frame rate per second. Therefore, possibility of industrial process monitoring and visualisation was also tested. At the beginning, calculations on a single computer were performed and at most 10–15 iterations of Landweber’s algorithm in one second have been achieved using single computer with CPU. In comparison, the KISDC platform (using two high performance nodes with multiple GPUs each) allowed for the reconstruction of 8 frames per second with 50 iterations of Landweber’s algorithm at the same time. Alternatively, if better image quality is demanded, it is possible to use highest possible image size that was tested (672KiB) and achieve 1 frame of image with 400 iterations of Landweber’s algorithm per second (Figure 13).

The system is flexible and can be extended by the next computing nodes, according to the needs.

## 6. Conclusions

A flexible, distributed computing system for tomographic image reconstruction called KISDC has been designed and developed. The system’s framework allows to accelerate any kind of computation dealing with a basic linear algebra operations. However, it should be noted that the KISDC is highly scalable and can be easily extended either by specific OpenCL kernel or by a plugin providing support for a special kind of calculations.

The work described in this paper was focused on improvement of data management in the distributed system and on reducing delays in the data transmission over the computer network. An original algorithm was designed that determines an array of performance coefficients for computing nodes in experimental way and allows to optimize the utilization of the system. The coefficient values are updated according to the current hardware configuration and to the system working conditions.

Also, a new network layer has been implemented. The comparison of times of data transfer and communication between the nodes shows very clearly that the use of the new developed system with HTTP protocol ensures much better results than with the Xgrid platform. It is also evident that the KISDC system allowed for a significant reduction of the total time of a single frame reconstruction and a major speed up in implementations of both the LBP and the Landweber’s algorithms.

## Abbreviations

The following abbreviations are used in this manuscript:

3D	three dimensional
API	Application programming interface
CPU	Central processing unit
ECT	Electrical capacitance tomography
FPGA	Field-programmable gate array
FTP	File transfer protocol
GPU	Graphics processing unit
HTTP	Hypertext transfer protocol
KIS	Computer engineering department (in Polish: *Katedra Informatyki Stosowanej*)
KISDC	KIS digital computing
LAN	Local area network
LBP	Linear back projection
RAM	Random access memory
SMB	Server message block
